# Local Economic Conditions Affect *Aedes albopictus* Management

**DOI:** 10.1007/s10393-024-01682-x

**Published:** 2024-04-24

**Authors:** Jacopo Cerri, Chiara Sciandra, Tania Contardo, Sandro Bertolino

**Affiliations:** 1 Dipartimento di Medicina Veterinaria, Università degli Studi di Sassari, Via Vienna 2, 07100 Sassari, Italy; 2 Research Centre for Plant Protection and Certification (CREA-DC), Florence, Italy; 3 Dipartimento di Ingegneria Civile, Architettura, Territorio, Ambiente e di Matematica, Università degli Studi di Brescia, Via Branze 43, 25121 Brescia, Italy; 4 Dipartimento di Scienze Della Vita e Biologia dei Sistemi, Università degli Studi di Torino, Via Accademia Albertina 13, 10123 Turin, Italy

**Keywords:** Tiger mosquito, Mosquito control, Arbovirus, Inequalities, Invasive species

## Abstract

Invasive mosquitoes are an emerging public health issue, as many species are competent vectors for pathogens. We assessed how multiple environmental and socio-economic factors affected the engagement of municipalities in Italy (*n* = 7679) in actions against *Aedes albopictus*, an invasive mosquito affecting human health and well-being, between 2000 and 2020. We collected information about mosquito control from official documents and municipal websites and modeled the role played by multiple environmental and socioeconomic factors characterizing each municipality through the random forest algorithm. Municipalities are more prone to manage *A. albopictus* if more urbanized, in lowlands and with long infestation periods. Moreover, these variables are more predictive of management in municipalities with a high median income and thus more economic resources. Only 25.5% of Italian municipalities approved regulations for managing *A. albopictus*, and very few of them were in Southern Italy, the most deprived area of the country. Our findings indicate that local economic conditions moderate the effect of other drivers of mosquito control and ultimately can lead to better management of *A. albopictus*. If the management of invasive mosquitoes, or other forms of global change, is subjected to local economic conditions, economic inequalities will jeopardize the success of large-scale policies, also raising issues of environmental and climate justice.

**Supplementary Information:**

The online version contains supplementary material available at 10.1007/s10393-024-01682-x.

## Introduction and Purpose

Invasive mosquitoes are an emerging public health and ecological issue. Many species are competent vectors for pathogens that undermine human health (Huang et al., 2019), and for which no medical treatment is available. Many countries are already experiencing the spread of invasive mosquitoes (Manguin and Boëte, 2011), but in the future their global distribution is expected to further increase, due to a synergy between climate change, that will make temperate regions suitable for tropical species (Iwamura et al., 2020; Ryan et al., 2019), and global connectivity, which promotes accidental introductions (Hulme, 2021).

In response to these scenarios, many researchers and practitioners called for adequate policies to manage invasive mosquitoes, at least where their impacts are the highest and their control is feasible (e.g., Europe; Bellini et al., 2020; Martinou et al., 2020). These could be based on multiple approaches targeting mosquitoes during their larval or adult phase (McGraw et al., 2013; Bonizzoni et al., 2013), which are more effective when used synergistically and coupled with the engagement of local communities (WHO, 2004*)*. However, despite some local successes (Muzari et al., 2017; Trewin et al., 2017), management attempts to prevent the spread of invasive mosquitoes, or to remove them from large spatial scales, were largely unsuccessful.

While this failure has been explained in terms of the decreasing effectiveness of chemical compounds, the lack of community mobilization, organization, engagement and empowerment (Ledogar et al., 2017; Dusfour and Chaney, 2022), the role played by policymakers’ engagement and how this is affected by structural barriers (Herring, 2010; Moise et al., 2018), has never been studied quantitatively. This gap is surprising, because the importance of local economic conditions and education for vector-borne diseases is now acknowledged (Adams et al., 2020; Morales-Pérez et al., 2017; Quintero et al., 2009; Spiegel et al., 2007) and because policymakers’ response depends upon human capital and available funding (Khan, 2016). Addressing this gap would be particularly urgent for local policymakers, as these are usually on the frontline of mosquito management. In this research, we studied how municipalities in Italy (*n* = 7679) engaged in the control of *Aedes albopictus*, a widespread invasive mosquito with relevant impacts on human health, between 2000 and 2020. *A. albopictus* is among the most successful invasive mosquitoes worldwide, due to its ecological plasticity and its capacity to adapt to urban environments and climate change (Kraemer et al., 2019; Liu-Helmersson et al., 2016). This species can host more than 20 different arboviruses and is a competent vector for those causing chikungunya, dengue and Zika (Gratz, 2004; McKenzie et al., 2019; Paupy et al., 2009).

In Italy, *A. albopictus* was introduced in 1990 (Sabatini et al. 1990) and it is now distributed in urbanized areas across most of the country, with infestation periods of several months in lowland and coastal areas (Pasquali et al., 2020; Petrić et al., 2021; Romi et al., 2008). Since 2007, *A. albopictus* was responsible for three epidemics of chikungunya in Italy. The first one was in 2007 and it involved the Emilia-Romagna region, with more than 200 cases (Rezza et al., 2007). Then, two more epidemics occurred in the Calabria (68 cases) and Lazio (approx. 330 cases) regions, during 2017 (Rezza et al., 2007, 2018; Vairo et al., 2018), which determined significant social costs (Trentini et al., 2018). Even though less severe, *A. albopictus* also has a non-negligible impact over human well-being by being a regular cause of bites (Caputo et al., 2020_;_ Carrieri et al., 2008). Its control, thus, became relatively common for Italian municipalities, mostly by banning water storage on private properties and by carrying out anti-larval treatments (Donati et al. 2020).

In many countries, local authorities report their engagement in mosquito control in official policy documents and websites: By collecting large sets of these documents, it is therefore possible to map mosquito control and link its occurrence to local factors, such as socio-economic and environmental conditions. Italian municipalities managing *A. albopictus* are obliged to authorize prevention and control measures, for example by approving dedicated regulations signed by the mayor. These official documents are available from the municipal websites, and thus, they allow to measure the engagement of local administrations in mosquito management, throughout the entire country at an extremely fine spatial scale.

## Methods

### Hypotheses and Selected Variables

Eight factors were selected to predict the engagement of municipalities in the management of *A. albopictus*: (i) local wealth, (ii) the duration of the infestation period and (iii) the invaded range in each municipality, (iv) the behavior of neighboring municipalities, (v) the average elevation, the quality of (vi) provincial and (vii) regional governance and (viii) the administrative region of each municipality.

Local wealth can be associated to three main factors affecting the management of mosquitoes. Since a good share of municipal budget in Italy comes from local taxation, a revenue which increases with the average municipal income (Bordignon et al., 2017), the public administrations in wealthier areas have more budget to hire private companies performing antilarval treatments. Due to long-term territorial inequalities, wealthy municipalities also have a larger and more educated/skilled staff, compared to poor ones (Viesti, 2023), which can understand the importance of mosquito control and engage into it. Finally, poor areas have more stagnating waters and vegetation due to residential abandonment, even in developed countries (e.g., North America, Little et al., 2017). Therefore, we hypothesized that municipal wealth is positively associated with the engagement of municipalities in the management of *A. albopictus* (Hypothesis_1_, H_1_).

In Italy *A. albopictus* occurs at many urban areas with a long infestation period (Pasquali et al., 2020; Petrić et al., 2021) that could also span the entire year (Romi et al., 2008). The duration of the infestation period represents the average number of days, in a whole year, with the presence of adult mosquitoes (Pasquali et al., 2020). Moreover, *A. albopictus* in Italy is regarded as a major disturbance by people (Caputo et al., 2020), especially at times when its population density peaks (Carrieri et al., 2008). Therefore, it is reasonable to assume that residents from those municipalities where *A. albopictus* is present for many months would experience prolonged levels of discomfort through biting, and municipal administrations would have a strong incentive to control mosquitoes, as citizen satisfaction could result into votes during municipal elections. Hence, we hypothesized that the duration of the infestation period is positively associated with the engagement of municipalities in the management of the species (H_2_).

In Italy *A. albopictus* is distributed almost entirely into urbanized environments (Caputo et al., 2020; Möhlmann et al., 2017), where it outcompetes other mosquitoes such as *Culex pipiens* (Marini et al., 2017), due to its faster larval development (Carrieri et al., 2003). The invaded range in each municipality was thus deemed to be the proportion covered by urbanized areas, the suitable habitat for *A. albopictus*. We hypothesized that the presence of *A. albopictus* would be more problematic for those municipalities with larger urban areas, where a higher proportion of the population is subjected to *A. albopictus* and it can push administrations toward managing the species. Thus, the probability of management should increase with the proportion of the invaded range (H_3_).

Engagement in mosquito control, like many other public policies, depends on its level of current adoption: For decision-makers, it is easier to engage in a policy when this is already implemented by their colleagues from neighboring areas (Pacheco, 2012). Therefore, we predicted that the number of neighboring municipalities who already engages in the control of *A. albopictus* is positively associated with the engagement of municipalities in the management of the species (H_4_). We quantified the number of neighbors by counting how many, among those municipalities who shared their boundaries with a certain municipality, engaged in the control of *A. albopictus*.

Almost certainly these 4 factors act synergistically, and economic availability is a major constrain: Even municipalities with long periods of infestation, or large urban areas where *A. albopictus* is common, are unlikely to engage in mosquito management if they lack the practical means to do so, such as funding or personnel. Therefore, we predicted that the magnitude of the association between municipal engagement and the duration of the infestation period (H_5_), the proportion of urbanized area (H_6_), and the number of engaging neighboring municipalities (H_7_), became more prominent for increasing municipal wealth.

Moreover, in Italy *A. albopictus* shows a marked altitudinal gradient, decreasing its abundance as elevation increases in both Central (Romiti et al., 2022) and Northern Italy (Baldacchino et al., 2017; Roiz et al., 2011). This pattern mostly depends upon climate change, which promoted the progressive diffusion of the species from lowlands to higher elevations, as increasing temperatures and changes in precipitation patterns could have reduced developmental and diapause times and increased adult survival and breeding sites (Romiti et al., 2022). Thus it is reasonable to assume that municipalities at higher elevations experienced a recent colonization by the species, and the population and local authorities still have a low awareness of the problem posed by *A. albopictu*s. Moreover, as *A. albopictus* does not perform blood feeding at temperatures below 15 °C (Marini et al., 2020), citizens of high-elevation municipalities would also experiment a reduced level of discomfort for most of the year, not pushing for mosquito control. This combination of reduced awareness and low discomfort led us to hypothesize that the probability of management would decrease with the increasing elevation of a municipality (H_8_).

Finally, we also hypothesized that the quality of governance of Italian Regions and Provinces should also be positively associated with engagement of municipalities in mosquito control. In Italy decision-making is a hierarchical process, and the behavior of municipalities is influenced by higher territorial units (https://ec.europa.eu/eurostat/web/nuts/background), such as provinces (NUTS-3) and regions (NUTS-2). Following and increased interest of the European Union regarding emerging diseases (e.g., Decision n. 2018/945), over the last years Italy developed and periodically updated a national plan for mosquito-borne diseases. The plan set up obligations for regional health authorities, about monitoring and containment. However, it did not provide any dedicated funding for the engagement of municipalities in mosquito control, leaving this task to regions, and then to provinces within each region.

In Italy deep differences in the quality of local governance were already found to affect sanitary policies (e.g., COVID-19 lockdown, Alfano and Ercolano, 2021). So, we hypothesized that regions and provinces with a higher value of the Institutional Quality Index (Nifo and Vecchione, 2015), were more effective in the implementation of the national plan for mosquito-borne diseases and therefore had municipalities that were more prone to engage in the control of *A. albopictus* (H_9_).

### Data Sources and Statistical Analyses

Data about engagement in *A. albopictus* control were collected from municipal official documents published between 2000 and 2020, across the entire national territory. As municipalities changed through time, due to merging or splitting, data were referred to 2020 municipal boundaries (*n* = 7,904), downloaded from the National Institute for Statistics (ISTAT, https://www.istat.it/it/archivio/222527). Municipal boundaries also indicated the mean elevation of each municipality. We searched for official documents in two steps. First, we queried Google using the name of each municipality and the following keywords in Italian: "municipal regulation *Aedes albopictus*" and "municipal regulation tiger mosquito(es)." Then, we queried the current and historical praetorian registers of each municipality by using "*Aedes albopictus* " and "tiger mosquito" as keywords.

Data from multiple years (2000–2020) were pooled into a dichotomous variable, indicating whether a certain municipality has ever done something for mosquito management. This choice was made because 31.1% of municipalities did not provide adequate information about the time and duration of their actions and any longitudinal analysis would have most likely been biased. Moreover, some municipalities authorized multi-year mosquito control on a single occasion. This approach did not allow us to perform any spatio-temporal modeling, capturing for example changes in mosquito control due to chikungunya outbreaks (Rezza, 2007, 2018; Vairo et al., 2018).

Local wealth was measured as the median income of residents in each municipality, in 2019. This simple metric was chosen because it is relatively stable in time and it allowed us to cover the entire country, being calculated for all municipalities. Data were obtained from the website of the Ministry of Economy and Finance (https://www1.finanze.gov.it/finanze3/pagina_dichiarazioni/dichiarazioni.php). Supplementary analyses indicated that the median income of residents, multiplied per the number of taxpayers, correlated well with total municipal revenues from taxation in 2019 (Fig. S1).

The duration of the infestation period was obtained by averaging predictions from a previous study (Pasquali et al., 2020). Notably, from a 1 km grid expressing the number of days of infestation for the whole national surface, we calculated the arithmetic mean for each municipality. The proportion of urbanized areas in each municipality was calculated from the 2018 Corine Land Cover (https://land.copernicus.eu/pan-european/corine-land-cover/clc2018).

The quality of local governance was expressed through the Institutional Quality Index (Nifo and Vecchione, 2015), a composite indicator based on five elementary indexes (reflecting corruption, governance, regulation, law enforcement and social participation), which measures how effective Italian provinces and regions are at policymaking. We calculated the median of the index, between 2004 and 2019.

Moreover, for post hoc comparisons between the engagement in the management of *A. albopictus*, and the socio-economic deprivation of Italian municipalities (see the Discussion), we calculated a composite index following Caranci et al. (2010). The index quantified socio-economic deprivation by combining the proportion of the resident population with a lower level of education, the rate of families with a single parent, the unemployment rate, the proportion of families who paid a rent for their house and the number of residents per squared kilometer. The final index was obtained by the sum of each standardized indicator, with highest values indicating the most deprived areas.

Our final dataset included complete data about 7679 municipalities out of 7904. For 225 municipalities the median income and institutional quality index were missing.

To test for our hypotheses, we adopted the Breiman’s random forest algorithm (Breiman, 2001), which aggregates classification trees, as building blocks to predict a response variable, according to a set of covariates (James et al., 2021). We preferred the random forests algorithm due to its predictive performances, its flexibility at discovering nonlinear interactions between covariates and its robustness against spatial correlation among neighboring areal units.

In our case, we predicted the probability that municipalities engaged in the management of *A. albopictus* in function of the median municipal income (H_1_), the length of the infestation period (H_2_), their proportion of urbanized surface (H_3_), their number of neighboring municipalities who already engaged (H_4_), their elevation (H_8_) and the institutional quality of their Region and Province (H_9_). We also included four interactions between median income, and the duration of the infestation period, the proportion of urbanized surface and the number of neighboring municipalities to test for H_5_-H_7_, respectively. We also included the latitude and longitude of each municipality, to detect large-scale geographical trends (Plant, 2018). Moreover, we added regions as covariates, to account for sources of variability in the data that did not depend upon institutional quality.

Overall model accuracy was quantified through the area under the curve (AUC). The relative importance of covariates was measured by averaging their rank according to three measures of importance: the mean decrease in model predictive accuracy and the mean decrease in the Gini index of node impurity after their permutation. Random forests were fitted with the “randomForest” package (Breiman et al., 2018) of the statistical software R (R Core Team, 2023).

## Results

**Fig. 1:**
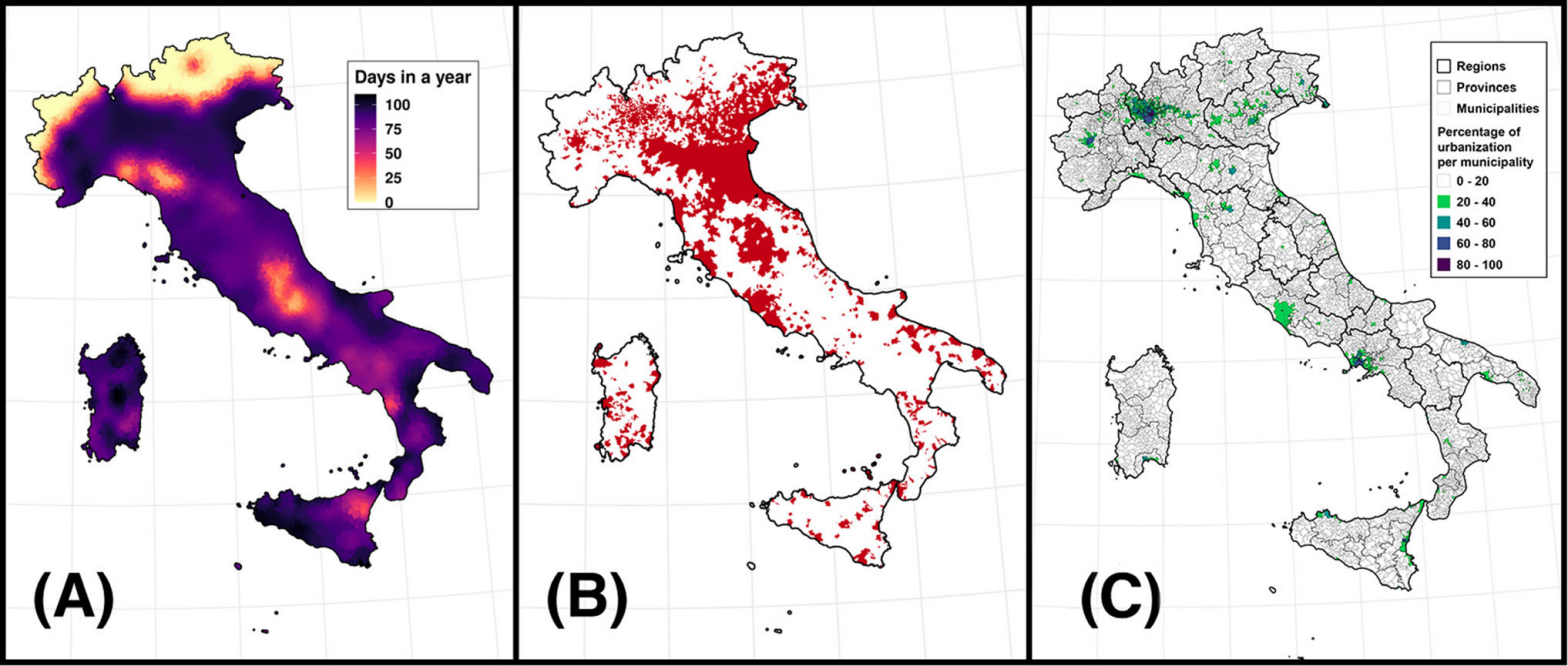
Presence and management of *A. albopictus* in Italy. Length of the infestation period in days (**A**), map of the municipalities in Italy that managed the species between 2000 and 2020 (**B**, in red), boundaries of regions, provinces and municipalities with the level of urbanization in each municipality (**C**) (Color figure online).

Overall, 2018 municipalities in Italy (25.5%) engaged in the management of *A. albopictus* between 2000 and 2020. They were located mostly in Northern and Central Italy, and management was almost absent from many regions in Southern Italy, even in areas characterized by long periods of infestation from *A. albopictus* (Fig. 1). Indeed, official documents that certified municipal engagement increased over time, especially after 2015. However, this increase had a much larger magnitude in Northern than in Central and Southern Italy (Fig. S2).

The random forest algorithm had a good classification accuracy on our test dataset, with an AUC of 0.86 ± 0.04 (median ± standard deviation) and an out-of-bag error of 0.15 ± 0.01. The number of neighboring municipalities which managed *A. albopictus,* the percentage of urban surface in each municipality, its elevation and the median income of each municipality were the most important covariates affecting prediction accuracy (Fig. S3). The region where municipalities were located was an important predictor whose effect was nevertheless highly variable across different regions (Fig. S4).

**Fig. 2:**
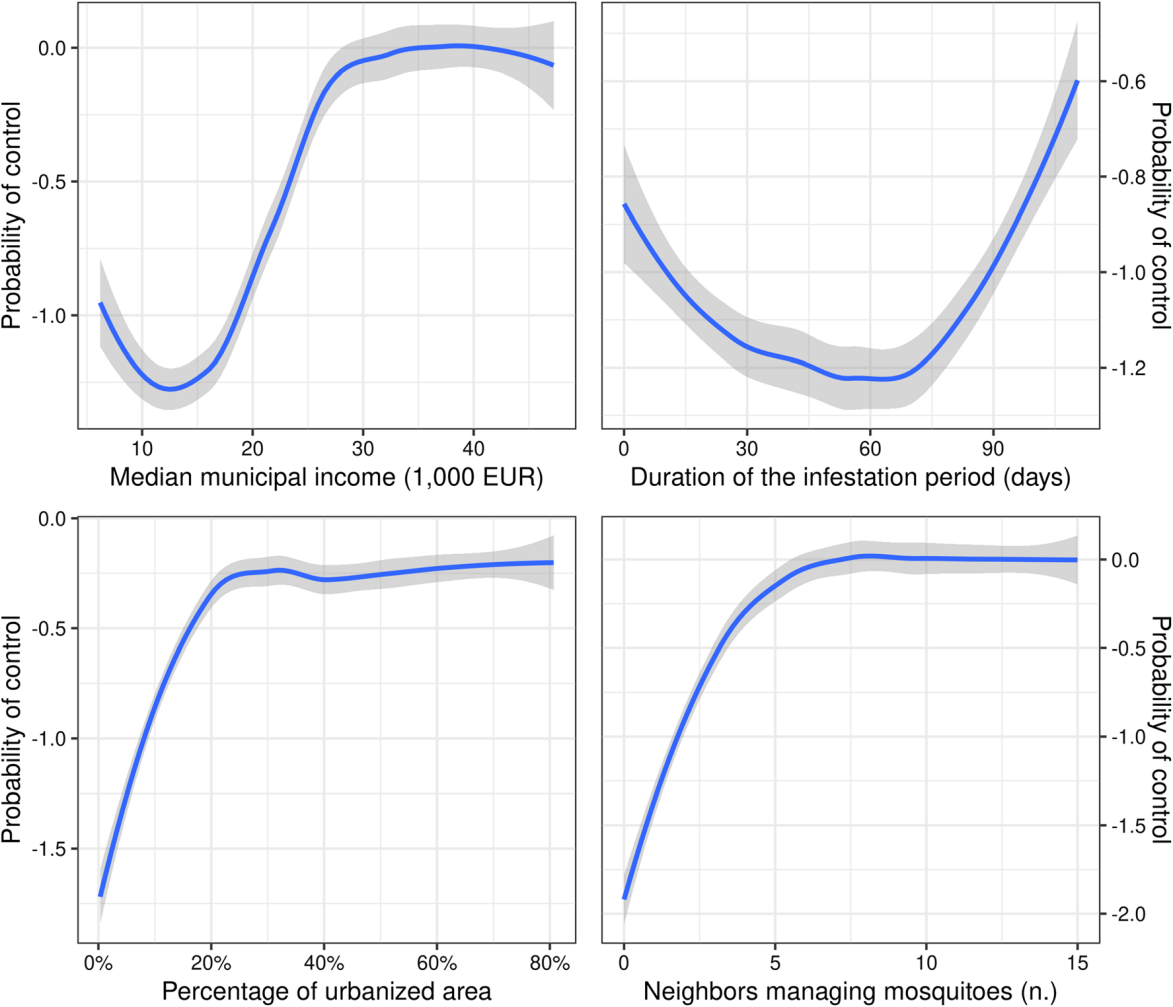
Partial dependence plots, showing the probability that municipalities engaged in the management of *A. albopictus*. Lower values on the *y*-axis indicate a lower probability of control, on the logit scale. Raw curves have been smoothed through a LOESS regression.

The probability of municipal engagement increased nonlinearly with median per-capita income, after 15,000 EUR per-capita. Moreover, it increased linearly with the length of the infestation period, for periods longer than 60 days. The probability of municipal engagement also increased nonlinearly with the proportion of urbanized surface, especially until 25% of the municipal surface was urbanized. The probability of municipal engagement also became progressively higher until around 5 of the neighboring municipalities engaged in the management of *A. albopictus* (Fig. 2).

**Fig. 3:**
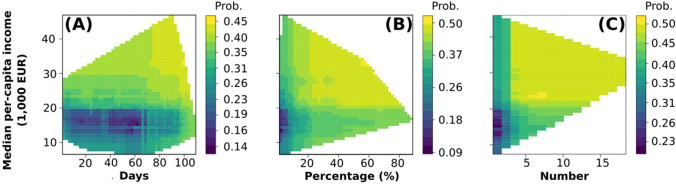
Interactive effect between median municipal income, and other covariates in the model, over the probability that municipalities managed *A. albopictus*: median municipal income and the length of the infestation period (**A**), percentage of urbanized area (**B**) and number of neighboring municipalities that managed *A. albopictus* (**C**). Lighter areas indicate municipalities with a higher probability of engagement, while darker areas indicate municipalities with a lower probability. Lower values on the y-axis indicate a lower probability of control, on the logit scale.

Bivariate partial dependence plots revealed a clear interaction between the median per-capita income, the length of the infestation period, the proportion of urbanized surface and the number of neighboring municipalities (Fig. 3).

The probability of municipal engagement decreased steadily up to an elevation of about 700 m (Fig. S5). Moreover, regions and provinces with a high quality of governance, were more prone to engage in mosquito control (Fig. S6).

## Discussion

At a time of global change, it is increasingly important to understand how policymakers deal with emerging ecological and public health issues, such as invasive mosquitoes, and how their decisions are modulated by practical constraints, such as local economic conditions. This is even more important at a time of pandemic. While COVID-19 clarified that human health and the environment are intertwined (Gillespie et al., 2021), it also had dire consequences for the global economy. Even those policymakers who would normally commit to manage global change will face limited economic availability and budget cuts (Pacheco, 2012).

This study shows that economic conditions are highly predictive for the management of emerging diseases and their impacts, even in developed countries, and that public policies addressing these issues could not ignore their economic context. Although some expert-based studies suggested that budget limits and constrained human resources could limit policies for mosquito control (Herring, 2010; Moise et al., 2018), to the best of our knowledge, this research was the first to explore this topic at a large geographical scale, using extremely fine-grained data and quantitative analyses.

Our findings indicate that Italian municipalities manage mosquitoes more often if they are urbanized (H_3_) and located in lowlands (H_8_), if many of their neighboring administrations do the same (H_4_), and if they are wealthy (H_1_). However, these results also indicate the absence of a proactive control strategy in Italy, since mosquitoes are controlled in areas with a more extended infestation period (H_2_), and not before they become well-established. We can conclude that local authorities frequently neglect the problem until the local well-being is affected, reacting to pressure from public health authorities and society. It is also not clear if the effect of neighboring municipality is due to natural coordination between neighboring administrations or if it is more based on an “administrative contagion” (Kavousi et al., 2020).

Moreover, there is a strong variation in the probability that municipalities from different regions engaged in mosquito control. Although municipalities from regions/provinces with a better local governance were slightly more prone to control mosquitoes (Fig. S6), those for Emilia-Romagna and Veneto regions had a much higher chance (Fig. S4). This could have depended upon past epidemics, like those of chikungunya in 2007 (Rezza et al., 2007), which led Emilia-Romagna and Veneto regions to develop effective communication channels with municipalities. This choice, in turn, probably made municipalities prone to engage in mosquito control during the following years.

We also believe that our study, based on observational data and a machine learning approach, could potentially suffer from unobserved confounding. Namely, unobserved characteristics of the resident population in a certain municipality (e.g., age) could influence both its decision to manage mosquitoes and local economic conditions. Future studies, based on real-time measurements of policymaker behavior (e.g., longitudinal surveys) and spatio-temporal modeling, or quasi-experimental approaches could be important to deal with unobserved confounding. Moreover, future studies should explore the extent to which local economic conditions might affect the distribution and accessibility of private properties, which is a limiting factors affecting the control of many invasive alien species, including mosquitoes (Bertolino et al., 2021).

**Fig. 4:**
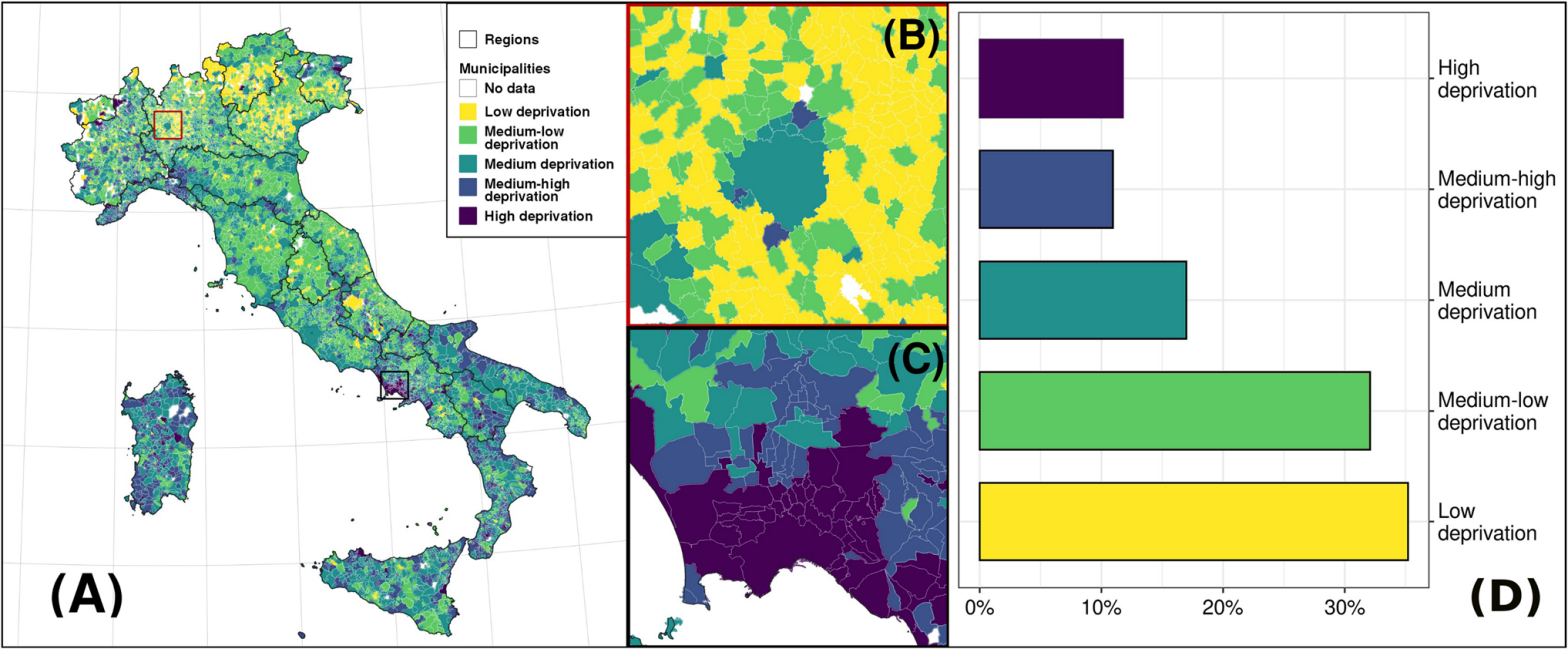
Spatial distribution of economic/material deprivation in Italy and its relationship to the management of *A. albopictus*. Left panel (**A**), darker areas are municipalities with the highest economic/material deprivation, light areas with the lowest one. In the middle, panel **B** highlights the city of Milan and surrounding municipalities and panel **C** Naples and surrounding municipalities. Right panel (**D**) reports municipalities from different quintiles of the deprivation index on how often they manage *A. albopictus*, expressed as percentage.

Local wealth was among the most important predictors, and its interplay with other predictors of municipal engagement was evident. In fact, with similar characteristics, the municipality engagement became the most pronounced in areas with a high median income (H_5_–H_7_). Broadly speaking, we believe that this interaction reflects the importance of the economic availability in the challenges posed by global change: Local policymakers can handle challenges only when they have the practical means to do so. This aspect, while apparently trivial (Herring, 2010; Moise et al., 2018), was never tested empirically, neither in invasion biology nor in the management of invasive disease vectors. However, we believe it to be of the uttermost importance. If the management of invasive mosquitoes, or other forms of global change, is subjected to local economic conditions, economic inequalities will jeopardize the success of large-scale policies, also raising issues of environmental and climate justice. This clearly emerged from our analysis: *A. albopictus* is almost not managed in Southern Italy, the poorest part of the country (Fig. 4), despite climate conditions there already support its widespread and prolonged presence (Fig. 1). Furthermore, while 30% of the less deprived Italian municipalities engaged in mosquito management, only about 10% of the most deprived did so (Fig. 4). This means that those areas that already have the highest level of economic and material deprivation are further suffering from invasive mosquitoes, because they do not have economic conditions suitable for their management. Hence, they are at risk of disproportionately suffering from mosquito-borne diseases in the future (e.g., chikungunya; Tjaden et al., 2017).

These conclusions about the role of economic conditions in the management of invasive mosquitos are, in our opinion, absolutely generalizable to Europe. The European Union is characterized by pronounced territorial inequalities (Iammarino et al., 2019), and in line with our conclusions about *A. albopictus* in Italy, some of its poorer member states are already failing to implement provisions of the first European regulation about invasive alien species (Regulation (EU) No 1143/2014 https://ec.europa.eu/commission/presscorner/detail/en/inf_21_2743), eight years after its enter-into-force.

## Conclusions

From a territorial management viewpoint, this study wants to encourage the development of policies for global change that explicitly address territorial inequalities. If the management of invasive mosquitoes is jeopardized and confined to a few wealthy areas, mosquito-borne diseases will remain largely unaddressed with an overall high impact. On the opposite, any resource provided to those poor areas that currently strive to control invasive mosquitoes will be repaid in terms of health and well-being for all the population. This consideration is particularly relevant for Europe, which is facing the establishment of invasive mosquitoes and their associated diseases (Medlock et al., 2015; Schaffner et al., 2013), and where the weakest economies are subjected to austerity and brain draining. Austerity led to budget cuts and low turnout rates of personnel, with predictable adverse outcomes over environmental management (e.g., wildfires in Greece; Papathoma-Köhle et al., 2021). Brain draining, in turn, could reduce available skilled figures capable of translating sophisticated approaches, such as integrated vector management, into concrete policies (Arrieta et al., 2017). Ultimately, we believe that addressing territorial inequalities will also mean tackling these two points.

More broadly, with this research we want to encourage future studies about the interplay between economic conditions and the implementation of policies for global change. Although many studies have already linked socio-economic dynamics to expressions of global change, such as biological invasions (Hulme, 2021), changes in forest cover (Curtis et al., 2018) or zoonotic spillovers (Allen et al., 2017), we also need to understand which factors affect the response capacity of the society and institutions. This response is a fragile chain of development and implementation of environmental policies, involving many different actors. Only by understanding how this complex response works it will be possible to make it more effective, equitable and rapid. There is a lot to be gained from a similar effort, at a time of global change.

### Supplementary Information

Below is the link to the electronic supplementary material.

Fig. S1 Association between the total percapita income of Italian municipalities and their total revenuesfrom taxation. Both values are on a log scale. 583 × 362mm (72 × 72 DPI)
Supplementary file1 (TIFF 4976 kb)

Fig. S2 Number of municipalities that approved regulations for managing A. albopictus, in Norther,Southern and Central Italy. 583 × 854mm (72 × 72 DPI)
Supplementary file2 (TIFF 11731 kb)

Fig. S3 Average rank of variable importance, measured across 1,000 random forests. Ranks range between1 (most important predictor) and 10 (least important predictor).158 × 132mm (350 × 350 DPI)
Supplementary file3 (TIFF 11662 kb)

Fig. S4 Differences between the various Italian regions, in terms of the probability that their municipalitiesadopted regulations for the management of A. albopictus. Values are logit contributions, with values on theright representing higher probabilities.185 × 158mm (350 × 350 DPI)
Supplementary file4 (TIFF 16337 kb)

Fig. S5 Probability that a municipality engaged in the control of A. albopictus, based on its elevation.95 × 73mm (350 × 350 DPI)
Supplementary file5 (TIFF 3913 kb)

Fig. S6 Probability that a municipality engaged in the control of A. albopictus, based on the institutionalquality index of its province and region.185 × 73mm (350 × 350 DPI)
Supplementary file6 (TIFF 7615 kb)
