# Supplementary figures and images for: Local Economic Conditions Affect Aedes albopictus Management
Source: Ecohealth. 2024 Apr 24;21(1):9–20. doi: 10.1007/s10393-024-01682-x (PMC11127834; doi:10.1007/s10393-024-01682-x)

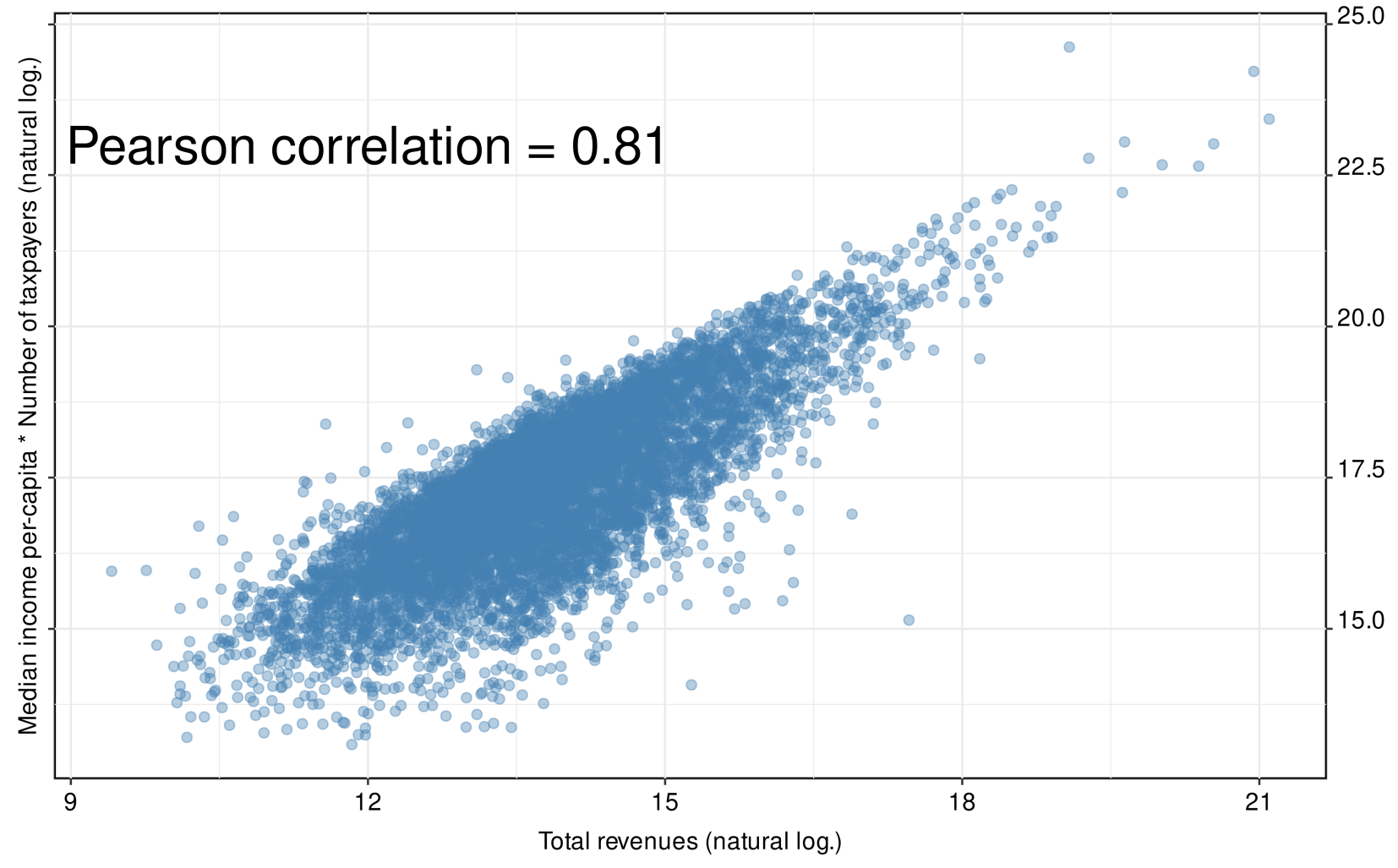

Supplement: Supplementary file 1 — Association between the total percapita income of Italian municipalities and their total revenuesfrom taxation. Both values are on a log scale. 583 × 362mm (72 × 72 DPI) Supplementary file1 (TIFF 4976 kb) [file 10393_2024_1682_MOESM1_ESM.tiff]

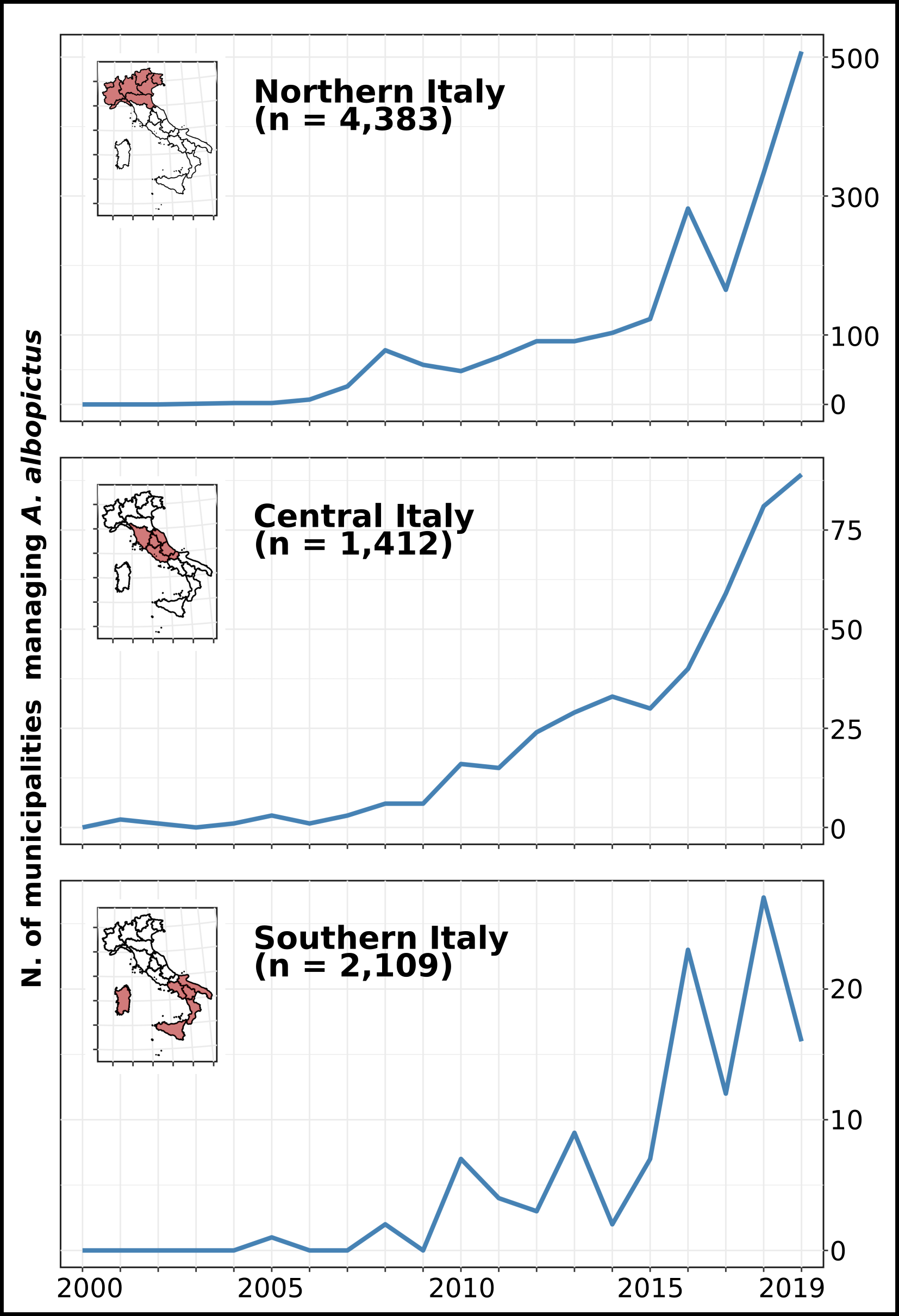

Supplement: Supplementary file 2 — Number of municipalities that approved regulations for managing A. albopictus, in Norther,Southern and Central Italy. 583 × 854mm (72 × 72 DPI) Supplementary file2 (TIFF 11731 kb) [file 10393_2024_1682_MOESM2_ESM.tiff]

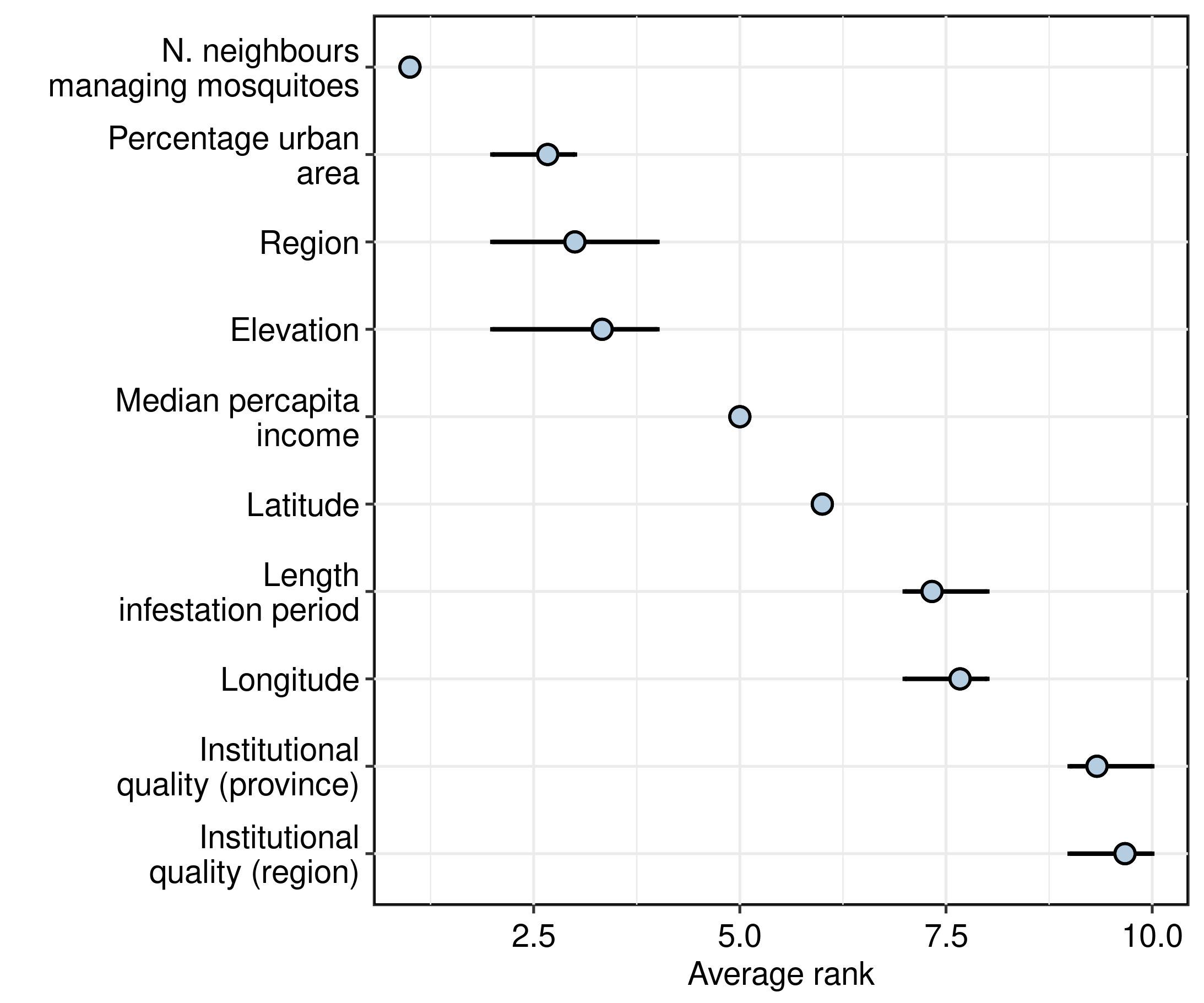

Supplement: Supplementary file 3 — Average rank of variable importance, measured across 1,000 random forests. Ranks range between1 (most important predictor) and 10 (least important predictor).158 × 132mm (350 × 350 DPI) Supplementary file3 (TIFF 11662 kb) [file 10393_2024_1682_MOESM3_ESM.tiff]

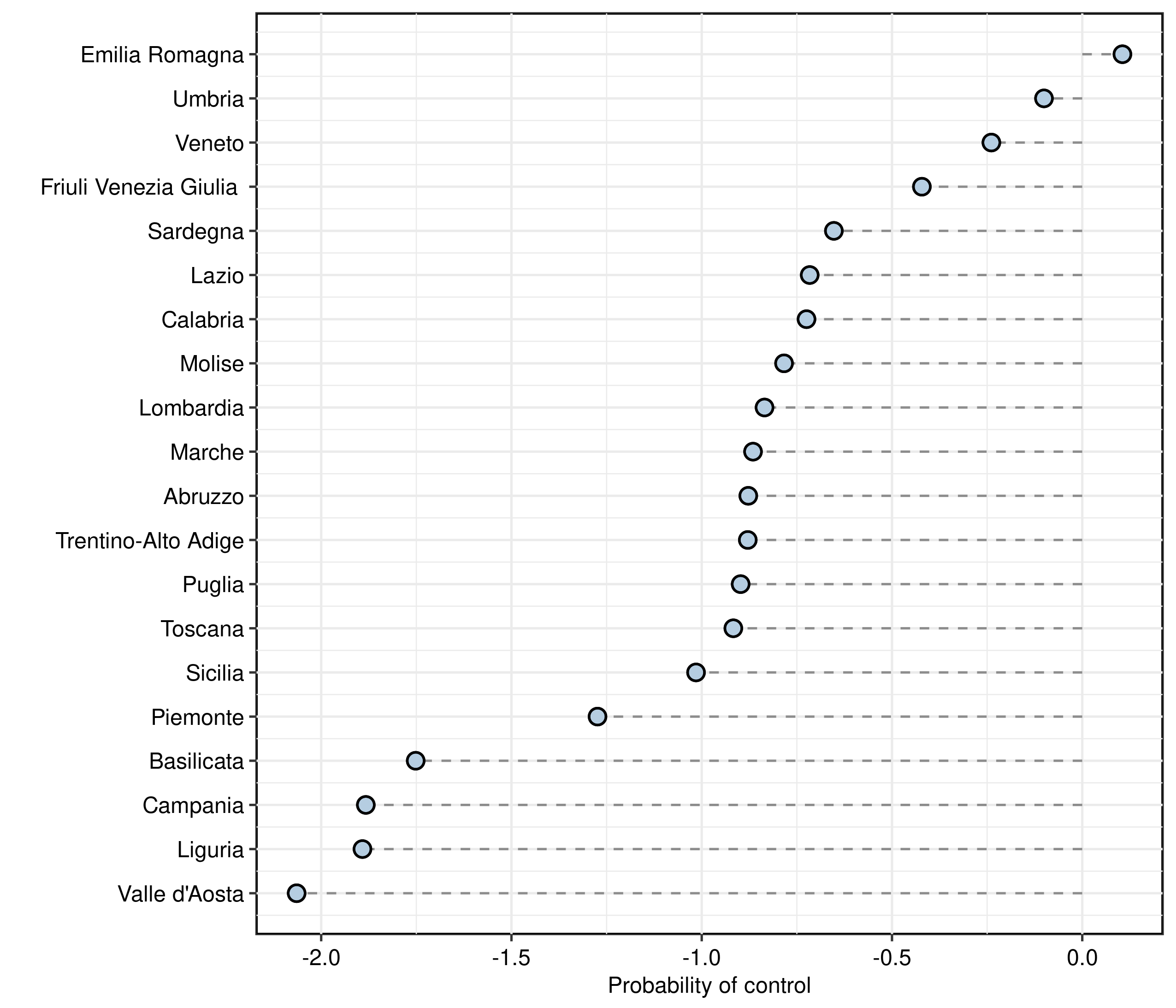

Supplement: Supplementary file 4 — Differences between the various Italian regions, in terms of the probability that their municipalitiesadopted regulations for the management of A. albopictus. Values are logit contributions, with values on theright representing higher probabilities.185 × 158mm (350 × 350 DPI) Supplementary file4 (TIFF 16337 kb) [file 10393_2024_1682_MOESM4_ESM.tiff]

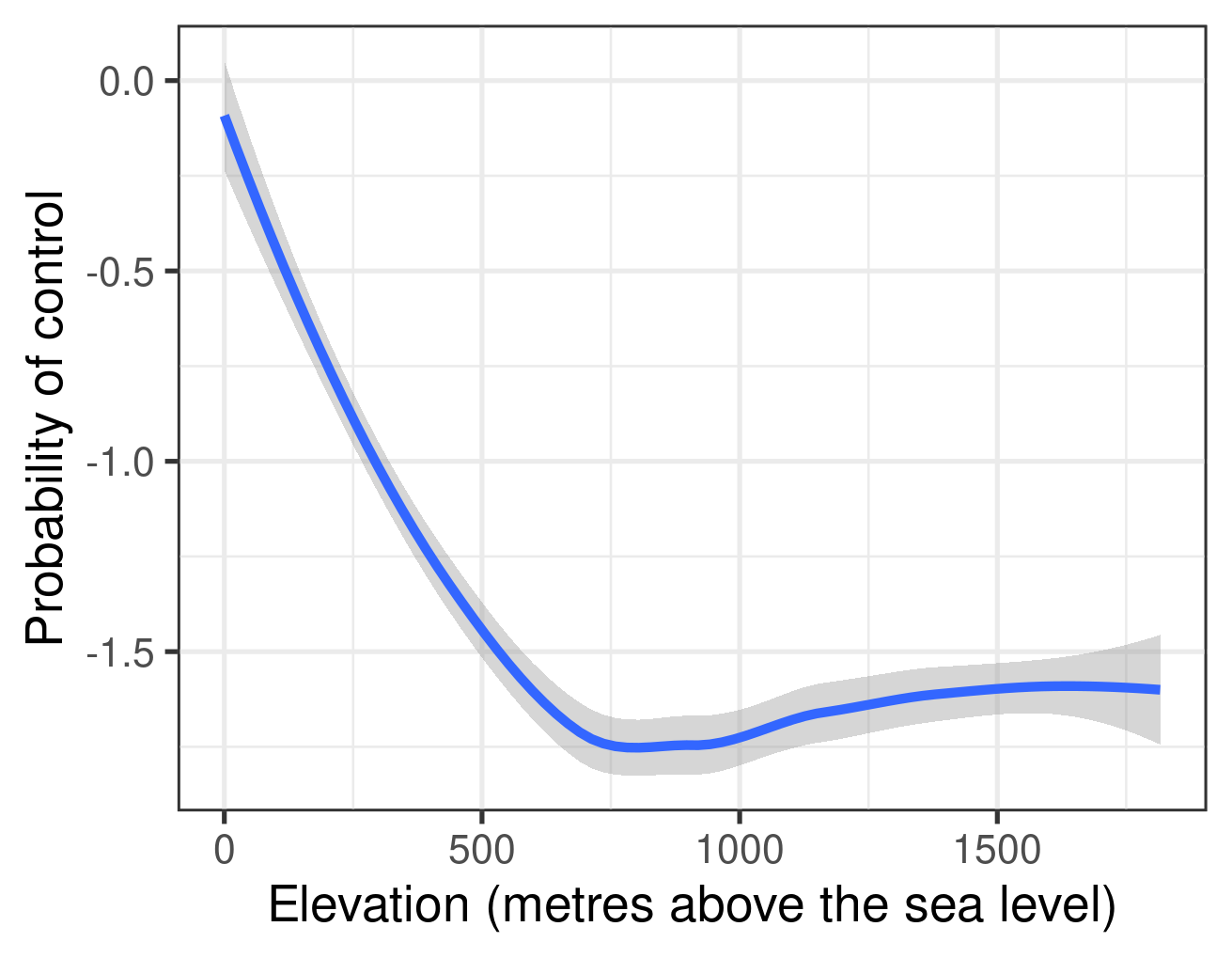

Supplement: Supplementary file 5 — Probability that a municipality engaged in the control of A. albopictus, based on its elevation.95 × 73mm (350 × 350 DPI) Supplementary file5 (TIFF 3913 kb) [file 10393_2024_1682_MOESM5_ESM.tiff]

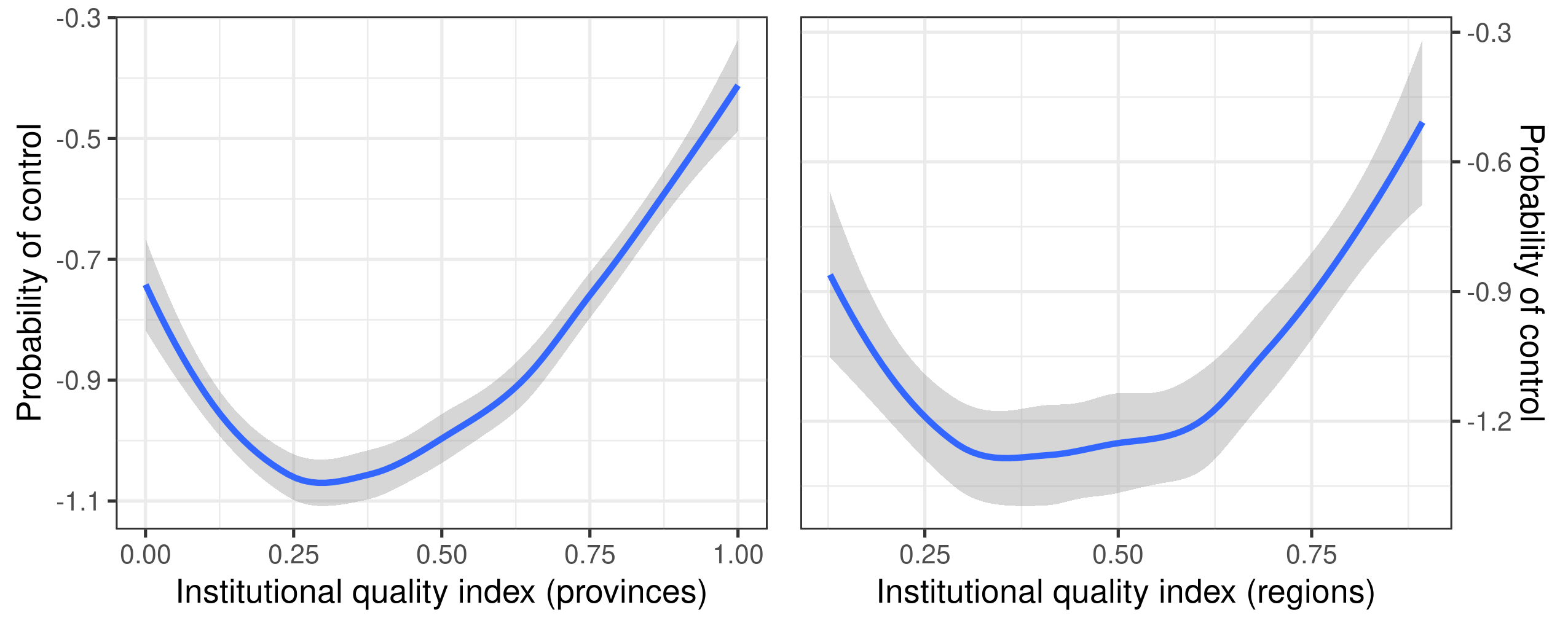

Supplement: Supplementary file 6 — Probability that a municipality engaged in the control of A. albopictus, based on the institutionalquality index of its province and region.185 × 73mm (350 × 350 DPI) Supplementary file6 (TIFF 7615 kb) [file 10393_2024_1682_MOESM6_ESM.tiff]
